# Can Any Good Thing Come out of Nazareth?

**Published:** 1874-08

**Authors:** 


Can any Good Thing Come out of Nazareth?—"We do not in any way wish to disparage our Western brethren, but it is a simple fact that by far the largest proportion of the leading minds of the profession are to be found in our Eastern cities."—*Philadelphia Medical Times.*

"I, of course, in common with all sensible people, have long since abandoned the idea that the ablest medical men are always to be found in the largest medical centres; for the history of this country, with that of all other countries, shows that very often the men who give to medical science its greatest improvements—whose genius is so great as to be impressed upon the medical history of the times—come from rural hamlets and small country towns. Velpeau, Magendie, Trousseau, Sir William Gull, Sir James Simpson, Sir Henry Thompson, in Europe; Warren Stone, Eve, Dudley, McDowell, Gross, Sims, and a host of others, in this country, all in their lives illustrate this fact."—*New York correspondent American Medical Weekly.*

